# Innovation in geriatrics: what this series means for care

**DOI:** 10.1093/geroni/igaf126

**Published:** 2025-11-11

**Authors:** Rama Chellappa

**Affiliations:** Department of Electrical and Computer Engineering and Biomedical Engineering, Johns Hopkins University Whiting School of Engineering, Baltimore, Maryland, United States

**Keywords:** Alzheimer's disease, Dementia, Artificial intelligence, Healthy aging

This supplement highlights advances in artificial intelligence (AI) and presents examples of the state of science and projects being developed at three Artificial Intelligence and Technology Collaboratories (AITC) for Aging Research—the Johns Hopkins AITC, the Massachusetts AI and Technology Center for Connected Care in Aging & Alzheimer’s Disease, and the Penn Artificial Intelligence and Technology at the University of Pennsylvania.

Older adults do best when care plans see the whole person. In the first article of this issue, Shang et al. (2025) show how bringing together clinical data with everyday social factors can improve risk spotting and care planning, moving beyond diagnoses to what actually drives health for each patient.

Home is where most care happens. The second article by Dolman et al. (2025) provides a systematic review that maps the most common unmet needs at home, medication management, mobility and safety, sleep, transportation, nutrition, technology usability, and care navigation, pointing to practical targets for services and tools that clinicians can act on today.

Any new technology should be age-friendly by design. The third article by Cudjoe et al. (2025) lays out a simple test: does it support What Matters, safe Medications, Mentation, and Mobility (4Ms), and is it easy to use, trustworthy, and built into real workflows? That roadmap makes it easier for teams to choose tools that actually help.

Where does AI stand right now? The fourth article by Abadir et al. (2025) offers a broad review that provides promise in monitoring, risk prediction, voice assistants, and assistive devices, but it reminds us that real-world value depends on reliable data, seamless integration, equity, and staff training. The clinical takeaway: ask whether a tool reduces workload, improves outcomes, and works for diverse patients.

In the fifth and final article, He et al. (2025) present a study that listens directly to family caregivers on social media. By analyzing a large set of posts, the team surfaces clear themes that clinicians will recognize: emotional strain, memory-related challenges, daily care tasks, family roles, and coping and support. These insights can guide brief counseling, targeted referrals (e.g., respite, memory care, caregiver support groups), and service design, meeting caregivers where they are.

In total, the articles in this supplement provide key takeaways for the field as it considers the role of AI: (1) pair whole-person data with age-friendly design, (2) choose tools that lighten the load, and (3) let caregivers’ voices shape care.

## Funding

This work was funded by the National Institute on Aging at the National Institutes of Health (P30AG073104).

## Conflict of interest

None declared.

## Supplement sponsorship

This article appears as part of the supplement “Engaging Older Adults and Families in AI and Technology Design,” sponsored by the National Institute on Aging at the National Institutes of Health (P30AG073104 to Johns Hopkins University, P30AG073107 to University of Massachusetts Amherst, and P30AG073105 to University of Pennsylvania).

## References

[igaf126-B1] Abadir M. , Dineen W., Myers D., Yu S., Phan P. (2025). Navigating the future of artificial intelligence technologies for improving the care of older adults. Innovation in Aging, 9(Suppl_1), S24–S32. 10.1093/geroni/igaf092 PMC12742854 41458888

[igaf126-B2] Cudjoe T. K. , Feemster K., Ashida S., Abadir P., Phan P., Schoenborn N. L. (2025). Age-friendly AI: Building bridges between technology and geriatric care. Innovation in Aging, 9(Suppl_1), S33–S37. 10.1093/geroni/igaf093 PMC12742840 41458887

[igaf126-B3] Dolman A. , Kaliappan S., Zhou Y., Palleti D., Marquard J., Lee S. I., Karkar R., Jimison H. B. (2025). A systematic review of unmet needs of older adults in home settings and their implications for novel technological solutions. Innovation in Aging, 9(Suppl_1), S14–S23. 10.1093/geroni/igaf106 PMC12742851 41458889

[igaf126-B4] He W. , Hou B., Zheng A., Feng Y., Klein A., O’Connor K., Yang S., Shang T., Demiris G., Gonzalez-Hernandez G. (2025). Advanced topic modeling with large language models: Analyzing social media content from dementia caregivers. Innovation in Aging, 9(Suppl_1), S38–S47. 10.1093/geroni/igaf120 PMC12742845 41458886

[igaf126-B5] Shang T. , Yang S., Zhai T., He W., Mamourian E., Zhang J., Hou B., Lee J., Moore J. H., Ritchie M. D., Shen L. (2025). A novel computational analysis integrating social determinants information from EHR and literature with Alzheimer’s disease biological knowledge through large language models and knowledge graphs. Innovation in Aging, 9(Suppl_1), S2–S13. 10.1093/geroni/igaf102 PMC12742847 41458885

